# The characterization of exosomes from fibrosarcoma cell and the useful usage of Dynamic Light Scattering (DLS) for their evaluation

**DOI:** 10.1371/journal.pone.0231994

**Published:** 2021-01-26

**Authors:** Tae Seong Lyu, Yoojin Ahn, Young-Jun Im, Seong-Soo Kim, Ki-Heon Lee, Jinyoung Kim, Yujin Choi, Dongwoo Lee, EunSeok Kang, Gayeon Jin, Jiwon Hwang, Sang-im Lee, Jung-Ah Cho

**Affiliations:** 1 College of Transdisciplinary Studies, School of Undergraduate Studies, DGIST, Daegu, Korea; 2 Department of New Biology, Graduate School, DGIST, Daegu, Korea; University of Hawai'i at Manoa, UNITED STATES

## Abstract

Exosomes are a type of extracellular vesicles containing mRNA, miRNA, and proteins of origin cells, which can control the characteristics of other cells or surroundings. Despite increasing evidence on oncogenic properties of tumor-derived exosomes, fibrosarcoma-derived exosomes remain largely unrevealed. While the proper extraction and characterization of exosomes is critical in exosomes research, there are various limitations in techniques to measure the size and homogeneity of exosomes. Here, we analyzed exosomes from a fibrosarcoma cell line WEHI-164 compared with a breast cancer cell line MDA-MD-231 as a control. Results from dot blot and western blot analysis demonstrated that GM1 ganglioside, and TSG101, HSC70 and GAPDH proteins were contained in exosomes from the WEHI-164 fibrosarcoma cell line. The existence of tetraspanins such as CD81, CD63 and CD9 was confirmed in the exosomes by ExoView analysis. The results obtained from TEM showed their sphere-like shapes of around 50 to 70 nm in radius. Through DLS, we found out that the mean radius of the exosomes derived from WEHI-164 and MDA-MB-231 cell lines was 94.4 nm and 107.8 nm, respectively, with high homogeneity. When comparing the radius measured by TEM with the radius measured by DLS, it was revealed that the difference between the two methods was about 40 nm. This study has significance in characterizing the molecular properties of exosomes from a fibrosarcoma, which has not been researched much before, and in providing clear evidence that DLS can be used as an efficient, convenient and noninvasive technique to simply check the homogeneity and size of exosomes.

## Introduction

Exosomes are 30–200 nm-sized extracellular vesicles (EVs) that are originated from multivesicular bodies (MVBs) in most cells. They contain various origin cell-specific intracellular molecules such as mRNA, miRNA as well as proteins, thereby playing a very important role as a molecular transport medium [1, 2]. Accumulating evidence suggests that exosomes can modulate not only the surrounding environment but also the characteristics of the cells around or at a distance [3–5]. Nevertheless, the need to understand the biological activity of exosomes is continuously increasing.

To better understand exosomes, it is important to maintain the quality of each preparation consistent and reproducible, so it is necessary to confirm and evaluate its quality each time. With increasing attempts to use exosomes in diagnostic and pharmaceutical applications as potential markers of human disease, accurate and objective control of EVs size and contamination after the purification process also becomes crucial [6–9]. So far, when studying exosomes, researchers have used dot blot, western blot, and electron microscopy (EM) as an initial confirmation process [10]. Among those, the process of identifying the form of exosomes with EM is time- and cost-intensive, and also requires a high level of device operation technology. Also, if the sample concentration is too high, the support film used in EM can be torn or damaged, so that the proper results would not be obtained.

Many attempts have been made to analyze EVs using various technologies before analyzing with EM. However, flow cytometry cannot detect the vesicles smaller than 300 nm, and atomic force microscopy (AFM) is time- and cost-intensive in imaging procedures. Nanoparticle tracking analysis (NTA) is fast, but requires large volumes and several optimization steps, and has low sensitivity in detecting vesicles less than 50 nm [6, 11]. Therefore, a more user-friendly, objective and relatively easy verification method is still needed.

In such an aspect, dynamic light scattering (DLS) is a potentially promising choice. Using lasers, DLS can analyze the velocity distribution of particle motion caused by Brownian motion via measuring the fluctuations of scattered light intensity and can calculate the particle size via the Stokes-Einstein equation [6, 12]. Since DLS is noninvasive and highly sensitive, it can be used to investigate a large number of vesicles at once and requires very little sample volumes, so that DLS had been used to analyze the size distributions of nanoparticles, exosomes, and liposomes [6, 12]. Furthermore, DLS does not require a pre-treatment process to dye or fix a sample, unlike EM, making it easier to know the size and homogeneity of the sample. Therefore, scientists who are researching about exosomes can simultaneously evaluate the quality and homogeneity of the exosome extract with the help of DLS.

Fibrosarcoma is a rare but highly malignant neoplasm of cells of the mesenchymal origin, such as fibroblasts in connective tissue. Although this type of cancer comprises approximately 10% of all sarcomas and its incidence has gradually decreased for several reasons, fibrosarcoma can invade the surrounding muscles or bones, and even spread throughout the body. Regarding their oncogenic properties, exosomes have recently attracted major attention in this field. However, exosomes from fibrosarcoma remain largely unknown. In this study, we characterized exosomes from fibrosarcoma using a mouse fibrosarcoma cell line WEHI-164 in comparison with previously well-characterized those from the MDA-MB-231 cell line, and compared DLS with EM as an exosome quality evaluation method.

## Methodology

### Cell culture and supernatant collection for exosome isolation

MDA-MB-231 [13], a human breast cancer cell line (Cat. #: 30026), and WEHI-164 [14], a mouse fibrosarcoma cell line (Cat.#: 21751), were purchased from the Korean cell line bank (KCLB). MDA-MB-231 and WEHI-164 cell lines were confirmed that they were not misidentified or contaminated according to the International Cell Line Authentication Committee (ICLAC) Database of Cross-contaminated or Misidentified Cell Lines. MDA-MB-231 were cultured with Dulbecco’s Modified Eagles Medium (DMEM) with High glucose (HyClone™ SH30243) containing 10% Characterized Fetal Bovine Serum (FBS) (HyClone™ SH30084). WEHI-164 were cultured with 10% FBS-containing RPMI 1640 media (HyClone™ SH30027) added with 25 mM HEPES (HyClone™ SH30337). For subculturing, we removed the culture media and washed with Dulbecco's Phosphate Buffered Saline (DPBS, HyClone™ SH30028), followed by adding 0.25% Trypsin-EDTA (Gibco® 25200114). The trypsinized cells were harvested by centrifugation for 5 min at 300 g and then counted manually by using trypan blue solution (SigmaAldrich, 72-57-1) and automatically with an automatic cell counter (JuLI^TM^ Br, NanoEntek). Cells were seeded on a T175 flask at the density of 3×10^6^ or 2×10^6^ cells for WEHI-164 or MDA-MB-231 cell lines, respectively. To eliminate any impact of exosomes in FBS on our results, we replaced the media containing 10% FBS with the media with 10% Exosome-Depleted FBS (Gibco™ One Shot™ LSA2720803) 48 hours before the cell culture supernatant was collected to extract exosomes.

### Exosome extraction and quantification

Exosomes from the culture medium were isolated by successive centrifugation. Briefly, the cell culture supernatant was successively centrifuged at 300 g for 5 min, at 1,200 g for 10 min, and at 10,000 g for 30 min at 4°C [2]. The centrifuged supernatant was concentrated by 100 kDa filter (Amicon® Ultra-15 Centrifugal Filter Unit UFC910024) and then finally ultra-centrifuged at 100,000 g for 1 hour to obtain the pellets of exosomes. After washing with DPBS by ultra-centrifugation at 100,000 g for 1 hour, we suspended the pellet in 100 μl of DPBS. To infer the quantity of isolated exosomes, protein assay was performed using the Pierce™ BCA Protocol Assay kit (Thermo Scientific™ 23227).

### Dot blot analysis

To assess the presence of an exosomal lipid GM1 ganglioside, we performed dot blot using HRP-conjugated Cholera toxin β subunit (CTxB, Sigma-Aldrich, C3741). We loaded 4 μg and 10 μg of whole cell lysate or exosomes on nitrocellulose (NC) membrane, air-dried and then added 5 ml of 5% normal goat serum (NGS) (abcam ab7481) to block the non-specific binding. After 1 hour, CTxB solution at 1:100 dilution with TBS was added to the membrane, which was then incubated at room temperature (RT) for 1 hour. After washing with TBST (Biosesang T2008) three times, ECL solution (Thermo Scientific™ 32106) was applied to the membrane, followed by visualization using illuminator (ATTO Luminograph II).

### Western blot analysis

Antibodies against TSG101 (1:200 dilution, Santa Cruz Biotechnology sc-7964), HSC70 (1:200 dilution, Santa Cruz Biotechnology sc-7298), Calnexin (1:1000 dilution, Santa Cruz Biotechnology sc-46669) and GAPDH (1:1000 dilution, Sigma-Aldrich ABS16 2930213) were used. We loaded 10~20 ug of whole cell lysate or exosomes in 10% resolving gels and electrophoresed in running buffer (1X Tris/Glycine/SDS Buffer) at 120 V for 1 hour. The resolved proteins were transferred to the NC membrane using the semi-dry transfer device (Bio-Rad Mini Trans-Blot® Cell) in transfer buffer (Biosesang TR2033) for 15 min. The membrane was then blocked with a blocking buffer (5% skim-milk in washing buffer) at RT for 1 hour. After three washes with washing buffer (0.1% Tween20 in TBS, Bio-Rad 1706531) for 5 min each, the primary antibodies described above were added to the membrane. After overnight at 4°C, HRP-conjugated secondary antibodies (1:5000 dilution, Merck #12–348; 1:2500 dilution, Thermo 31430) were added and incubated at RT for 1 hour, followed by three times of washing with washing buffer. The presence or quantity of each protein marker was analyzed using ECL solution and illuminator as used in the dot blot.

### ExoView analysis

The isolated exosomes were detected for CD81, CD63 and CD9 with ExoView platform (NanoView Biosciences, Boston, MA) according to the manufacture’s instruction. Briefly, the diluted exosomes were incubated overnight with Exoview tetraspanin Chip (EV-TC-TTS-01) arrayed with antibodies against CD81, CD63 and CD9. IgG isotype was used as a negative control. After washing, the chips were incubated with Exoview tetraspanin labeling ABs (EV-TC-AB-01) containing the fluorescence-tagged labeling antibodies against CD81, CD63 and CD9. Finally, the chips were imaged with the ExoView R100 reader using ExoViewer 2.5.5 acquisition software, and the data were analyzed using ExoViewer 2.5.0 with sizing thresholds set to 50 to 200 nm diameter. Normalized particles were counted as the differences between post-scan and pre-scan. The pre-scan value represents the number of fluorescently labeled antibodies without any extracted samples.

### Dynamic Light Scattering (DLS)

The extracted exosomes were diluted to 0.1 μg/μl using DPBS and then transferred into a cuvette, which was finally filled up to 100 μl with DPBS. After the laser and temperature equilibrium of the device (Dynapro Nanostar, WYATT Technology) was stabilized, we put the exosome-containing cuvette (NanoStar MicroCuvette Kit) into the DLS device to start measuring. For reproducibility and standardization, the parameter values were fixed as follows; Laser Wavelength (nm): 663.87, Temperature Controlled: yes, Peak Radius Low Cutoff(nm): 0.5, Peak Radius High Cutoff(nm): 5000, Auto-attenuation Time Limit(s): 60, Calculate D10/D50/D90: yes, Calculate Polydispersity: yes, Set temperature(C): 25, Wait (min): 5. The size or homogeneity of exosomes was recognized through the radius or the % Intensity, respectively. The measurement with DLS was conducted with 10 acquisition of 5 seconds. N represents the number of extract from each cell line, and n represents the number of data collected from each samples (WEHI-164: N = 7, n = 12, MDA-MB-231: N = 13, n = 16).

### Transmission Electron Microscopy (TEM)

Purified exosome preparations (5 ul) were diluted with an equal volume of 4% paraformaldehyde at 4°C for 30 min, and then carefully placed on a carbon-coated 300-mesh copper grid for 20 min, followed by fixed by 1% glutaraldehyde for 5 min. After washed two times, the grids were contrasted with 2% uranyl acetate and then washed again two times. The morphology of isolated exosomes was visualized with TEM (FEI/Tecnai G2 F20 TWIN TMP). N stands for the number of pictures and n stands for the number of exosomes in the pictures (WEHI-164 cell line: N = 26, n = 98, MDA-MB-231 cell line: N = 8, n = 12). The images of exosomes obtained from TEM were analyzed by ImageJ software to calculate the radius of exosomes.

### Statistical analysis

An F test was used to test whether the two populations had equal variances. After the F test, to test whether we can claim the equivalence between two data sets, a two-sample equivalence test was also used. The two sample t-test was conducted with a 95% confidence interval to test whether the radius of exosomes derived from MDA-MB-231 and WEHI-164 cell lines obtained from DLS and TEM had different means (*p<0.05, **p<0.001, ***p<0.0001). The F test and the two-sample equivalence test were conducted through minitab19, and the two-sample t-test was conducted through GraphPad Prism (GraphPad software).

## Results

### Validation of exosomes isolated from fibrosarcoma cell

**Fig 1:**
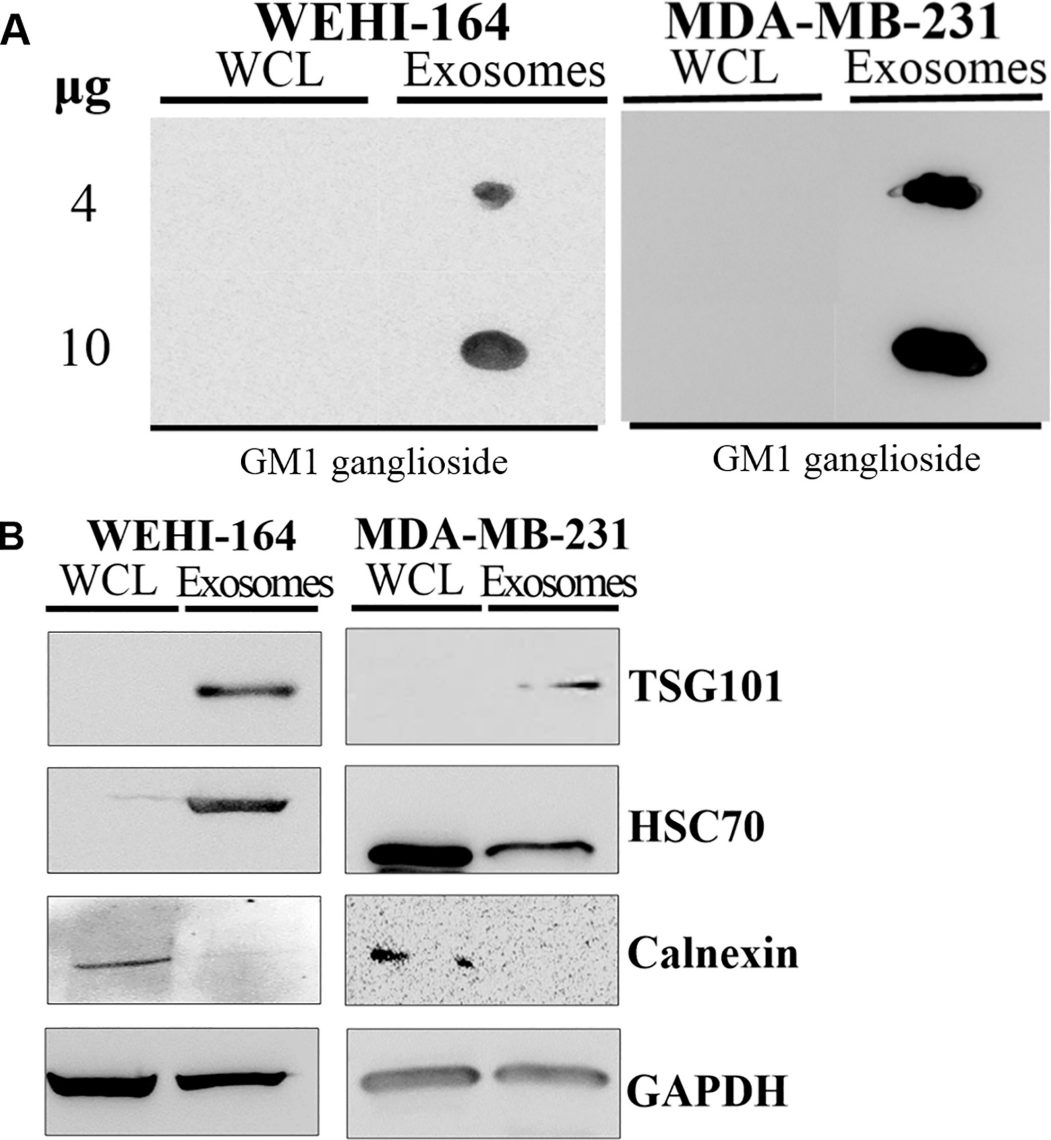
**(A)** GM1 ganglioside was detected in the exosomes extracted from the WEHI-164 cell line. The results of the dot blot to detect GM1 ganglioside using Cholera Toxin β subunit (CTx β) are shown. Dot blot was performed with 4 or 10 ug of the whole cell lysates (WCL) and extracted vesicles (Exosomes) from the WEHI-164 and MDA-MB-231 cell lines. **(B)** TSG101 and HSC70 were detected in the exosomes extracted from the WEHI-164 cell line. The results of the western blot for WCL and extracted vesicles (Exosomes) from the WEHI-164 and MDA-MB-231 cell lines are shown. In the WEHI-164 cell line, TSG101 and HSC70 were highly detected in exosomes, and calnexin was only detected in cells. Data from the MDA-MB-231 cell line showed similar results with the WEHI-164 cell line. GAPDH is used for normalization and positive control.

We investigated the exosomes from the mouse fibrosarcoma cell line for the marker molecules that were normally used for validation of exosomes. First, dot blot analysis was performed to detect the lipid components of exosomes. We used the Cholera Toxin β subunit (CTx β) because it was known to bind to GM1 ganglioside which is a lipid component of the plasma membrane and enriched in the exosomes [15, 16]. The result showed that GM1 ganglioside was detected proportionate to the concentration of exosomes of the WEHI-164 cell line as in the exosomes from the MDA-MB-231 cell line (Fig 1A). As expected, GM1 ganglioside was not detected in both whole cell lysates. This result demonstrated that exosomes from the WEHI-164 cell line contained GM1 ganglioside.

Secondly, western blot analysis was used to detect proteins that are specifically expressed or not resident in the exosomes. We detected TSG101 and HSC70 as the exosome-enriched protein markers, and Calnexin as the non-exosomal marker [17–19]. The result showed that exosomes from the WEHI-164 cell line contained highly enriched TSG101 and HSC70, whereas no detection of Calnexin (Fig 1B). Exosomes from the MDA-MB-231 cell line showed similar results, although HSP70 was not enriched as much as shown in the WEHI-164 cell line.

**Fig 2:**
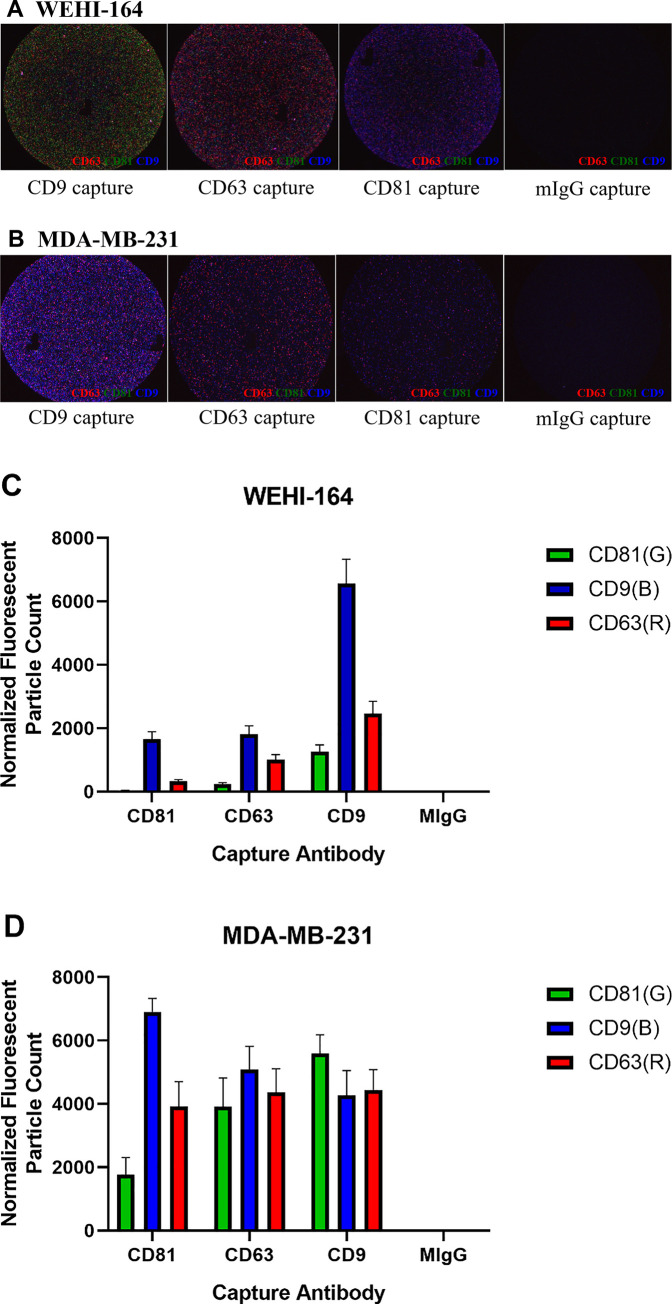
CD81, CD63 and CD9 were detected in the exosomes extracted from the WEHI-164 cell line. Using capture and detection antibodies, the levels of tetraspanins contained in the exosomes were investigated. Exosomes were primarily captured by each of CD9 or CD63 or CD81, and then fluorescently labeled for CD81 (green), CD9 (blue) and CD63 (red). (A, B) Representative images of immunofluorescence-labeled exosomes from the WEHI-164 (A) and MDA-MB-231 (B) cell lines for tetraspanins are shown. (C, D) Tetraspanin-positive exosomal vesicle counts are shown. Data represent the mean of six samples for the WEHI-164 cell line (N = 6, n = 18) and four samples for the MDA-MB-231 cell line (N = 4, n = 12) with a standard error of the mean (SEM), and each sample was performed in triplicate. CD9 was remarkably abundant in exosomes from the WEHI-164 cell line (C), while exosomes from the MDA-MB-231 cell line were mostly triple-positive for CD81, CD9, CD63 (D).

Thirdly, we investigated the expression of CD63, CD9 and CD81, which are the well-known tetraspanins to be enriched in the exosomes (Fig 2). Using Exoview affinity microarray platform analysis, exosomes were primarily captured by antibodies against each tetraspanin, and then fluorescently labeled by detection antibodies for the three tetraspanins (Fig 2A and 2B). The results revealed that the exosomes from both cell lines were all positive for the three tetraspanins. It was notable that the exosomes from the WEHI-164 cell line indicated enhanced fluorescence signal of CD9 than CD81 or CD63, while exosomes from the MDA-MB-231 cell line showed similarly high levels of the tetraspanins (Fig 2C and 2D).

### Morphology of exosomes identified using TEM

**Fig 3:**
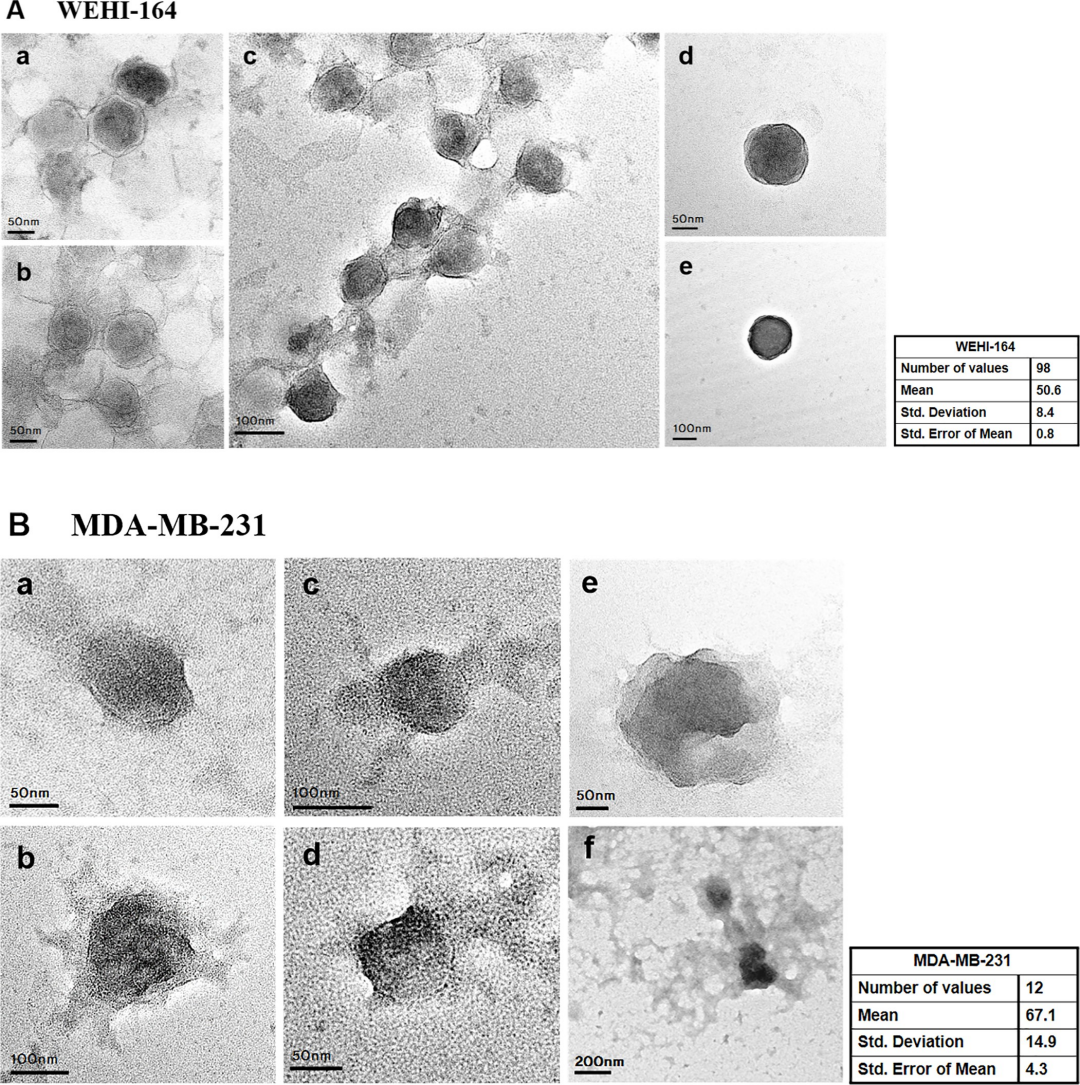
The shape and size of exosomes extracted from the WEHI-164 cell line were identified through TEM. Representative images for exosomes from WEHI-164 (A, N = 26, n = 98) and MDA-MB-231 (B, N = 8, n = 12) cell lines are shown. We verified that the isolated exosomes have the sphere-shaped morphology and were in the range of the expected size [17–19]. The scale bar represents (A-a, b, d, B-a, d, e) 50 nm, (A-c, e, B-b, c) 100 nm and (B-f) 200 nm, respectively.

Then, we verified the morphology and size of exosomes via TEM (Fig 3). The results confirmed the sphere-shaped vesicles with bilayer, which were the same as the previously known morphology of exosomes [20]. Also, we have identified that the mean radius of exosomes isolated from the WEHI-164 and MDA-MB-231 cell lines were 50.6 nm and 67.1 nm, respectively.

### Measurement of radius and homogeneity of the exosomes via DLS

**Fig 4:**
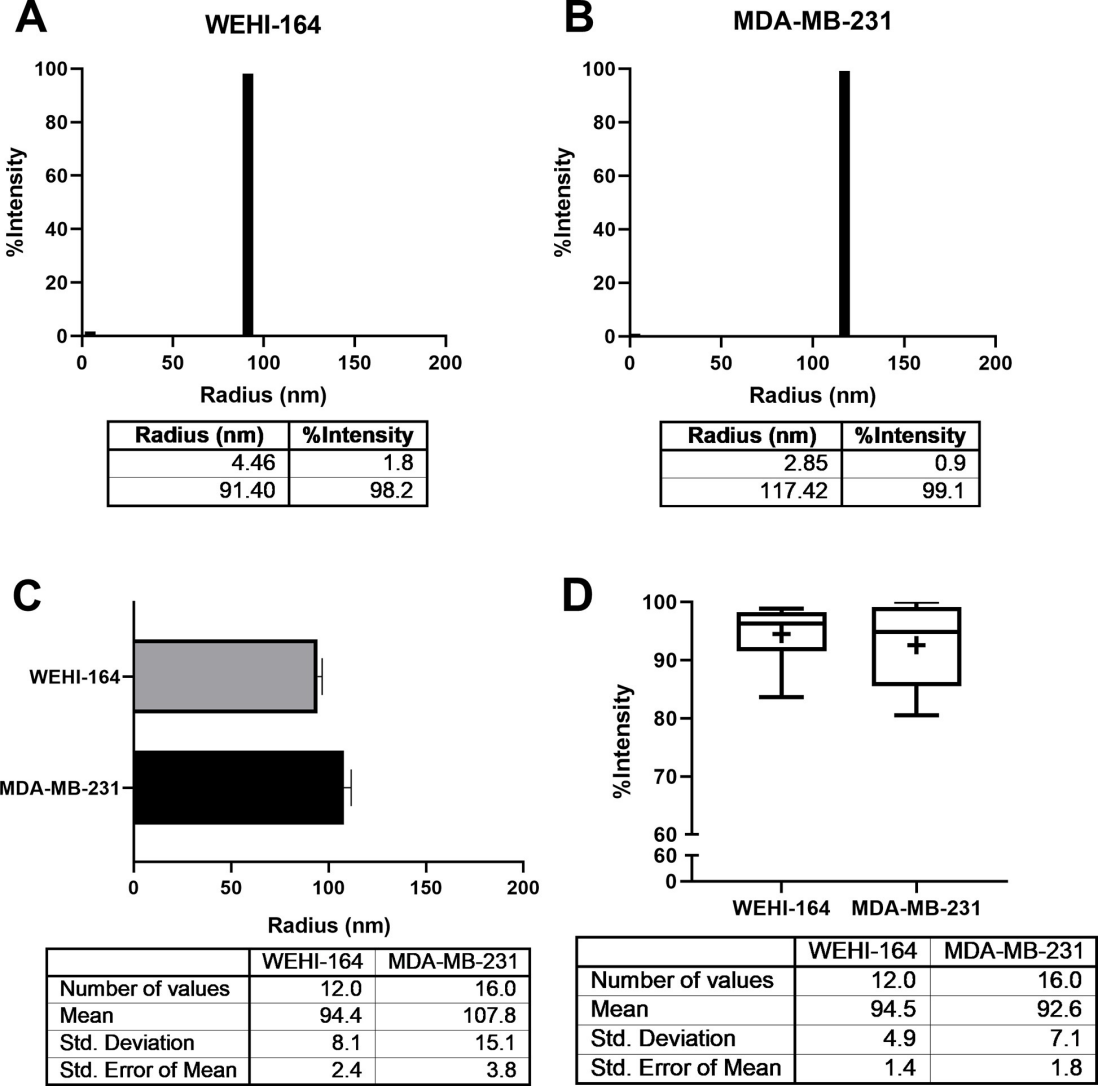
The average radius of exosomes derived from the WEHI-164 cell line was identified by DLS. (A, B) A representative DLS result for the size distribution of exosomes is shown. For each bar in the graph, the size and intensity were (4.46 nm, 1.8%) and (91.40 nm, 98.2%) for exosomes from the WEHI-164 cell line (A), and (2.85 nm, 0.9%) and (117.47 nm, 99.1%) for exosomes from the MDA-MB-231 cell line. (C) The mean radius of exosomes isolated from the WEHI-164 (94.4 nm from N = 7, n = 12) (gray bar) and MDA-MB-231 (107.8 nm from N = 13, n = 16) (black bar) cell lines are shown. Error bars represent the standard error of the mean. (D) The mean intensity of exosomes (sized in radius around 100 nm) isolated from the WEHI-164 (94.5%) or MDA-MB-231 (92.6%) cell lines is marked as **+**. The line in the box states the median of the intensity data. Error bars represent the maximum and minimum of data.

Finally, the exosomes were subjected to DLS (Fig 4). The measurement with DLS was conducted more than twelve times and the results were statistically analyzed. Since DLS presents the data on the size distribution and the intensity of each, we first analyzed the radius of the exosomes from size distribution. Although centrifugation steps are essential in the exosome isolation process, the size distribution data obtained after the high-speed(ultra) centrifugation should be interpreted with caution because high speed centrifugation may cause aggregation of EVs [21, 22]. Therefore, here we performed the re-suspension and re-centrifugation in DPBS vigorously and repeatedly so as to possibly remove the impurities and aggregations [7, 23]. For analysis, we used the radius data with the main peak which reached the highest intensity. When measuring the size, the exosomes derived from the WEHI-164 cell line between 80 nm and 100 nm in radius showed an intensity of more than 90%, while exosomes derived from the MDA-MB-231 cell line between 80 nm and 130 nm showed an intensity of more than 85%, the wider size range of exosomes of the WEHI-164 cell line. Taken together, it was revealed that the mean radius of exosomes from the WEHI-164 cell line was 94.4 nm, while those from the MDA-MB-231 cell line was 107.8 nm (Fig 4C). Next, we analyzed the homogeneity of exosomes with around 100 nm in radius from the intensity %. The result revealed that the mean intensity of exosomes isolated from the WEHI-164 or MDA-MB-231 cell lines was 94.5% or 92.6%, respectively (Fig 4D).

### The radius measured by DLS showed statistical significance with a difference of about 40 nm from that observed by TEM

**Fig 5:**
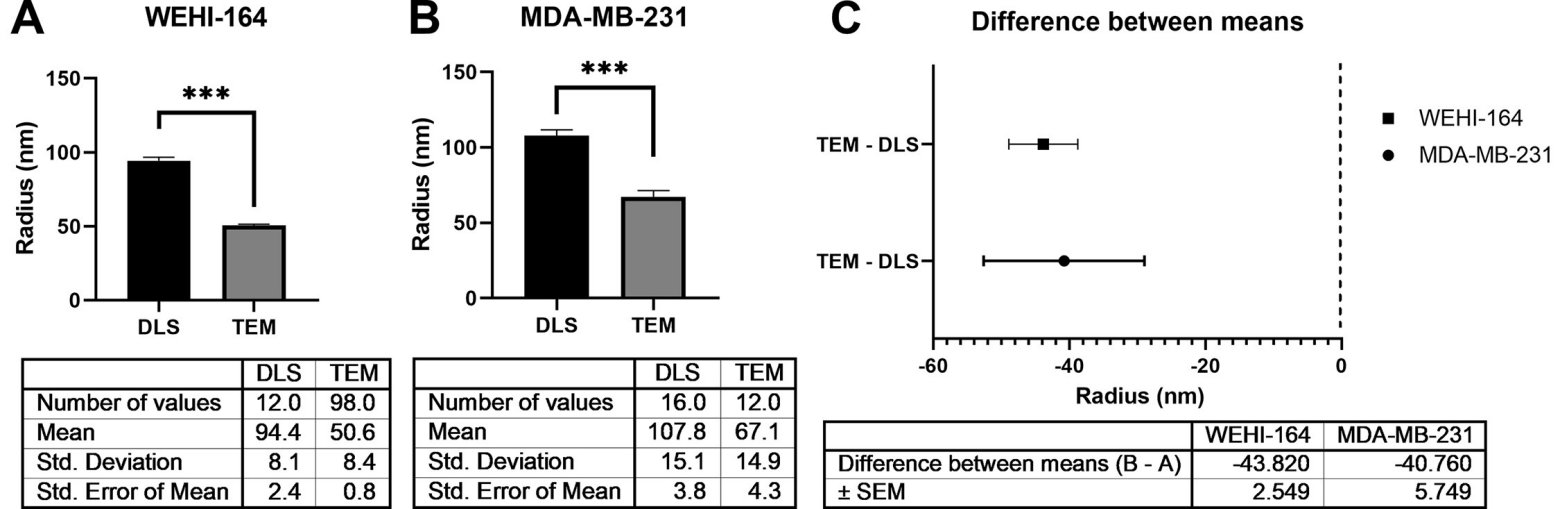
The mean radius of the exosomes from the WEHI-164 cell line measured by DLS and TEM was confirmed statistically significant with about 40 nm difference. The average radius (nm) of exosomes derived from the WEHI-164 (A) and MDA-MB-231 (B) cell lines measured by DLS and TEM is shown. The mean radius of exosomes from the WEHI-164 cell line was 94.4 nm and 50.6 nm by DLS (N = 7, n = 12) and TEM (N = 26, n = 98), respectively, while from the MDA-MB-231 cell line was 107.8 nm and 67.1 nm by DLS (N = 13, n = 16) and TEM (N = 8, n = 12), respectively. Statistical significance conducted by the two sample t-test with a 95% confidence interval is indicated as *** (p<0.0001). (C) The difference between the mean radius measured by TEM and DLS was represented. The difference between exosome sizes measured by DLS and TEM was about 40 nm in radius in both cell lines. Error bars represent the standard error of the mean.

Several statistical analysis methods were used to examine whether the population means of the radius measured by DLS and TEM were statistically comparable or not (Fig 5). Levene’s F test (α = 0.05) results showed that multiple comparison interval for the standard variation of both radius data measured by TEM and DLS was overlapped and had equal variances in the radius of exosomes extracted from the WEHI-164 (p = 0.0264) and MDA-MB-231 (p = 0.878) cell lines. Compared to radius data measured by DLS with TEM, the data of both cell lines could not claim the equivalences as a result of the two-sample equivalence test. Through the two-sample t-test and the two-sample equivalence test, respectively, we could confirm the statistical differences of the data of both cell lines obtained by DLS and TEM (Fig 5A and 5B). The absolute value of the difference between means (TEM-DLS) was about 40 nm in radius for both cell lines (Fig 5C), indicating that the radius measured by DLS was larger about 40 nm than by TEM.

## Discussion

We characterized exosomes from a mouse fibrosarcoma cell line compared with a human breast cancer cell line using DLS, TEM, ExoView, western blot and dot blot, and demonstrated that exosomes from fibrosarcoma contained GM1 ganglioside, HSC70, TSG101, tetraspanins such as CD9, CD81 and CD63, and mainly sized around 50~100 nm in radius. This finding is particularly significant in that we analyzed the properties of exosomes of mouse fibrosarcoma cell lines that were not previously studied well.

Clinically, fibrosarcoma is known to have a poor prognosis due to its high aggressiveness, low sensitivity to radiation and chemotherapy as well as high recurrence rates [24]. Furthermore, the current best treatment for fibrosarcoma is the removal by general surgery, suggesting the need for new treatment strategies. Given the importance of accurate diagnosis for proper therapy planning, this study has strong implications. Exosomes are the entities that contain molecules and also genetic information of their origin cells. After being secreted from cells, they may flow into the blood and can travel long distances in the body, so they can be used as a useful indicator for diagnosis or even for monitoring the prognosis during treatment by investigating their presence and contents. In order not to lead to false conclusions, the accurate identification of exosomes is the critical first step. Here, we provided the basic information about exosomes from fibrosarcoma. It is also of great value that this study presents the availability of DLS as a primary tool for the exosome investigation.

DLS 1) is available to obtain information about a large number of particles in a short period of time, 2) has noninvasive preparation steps, which do not require chemical fixation or labeling, thereby allowing the sample with complete recovery, 3) is highly sensitive, 4) has simplicity that let DLS user-friendly and accurate, 5) automatically adjusts the attenuator to measure the wide range of sample concentration, 6) is available for broad concentration range about 10^8^~10^12^ particles/ml, and 7) is less costly than other techniques [6, 12, 25, 26]. With these advantages, DLS has become the proper devices to determine the EVs size distribution for routine usage [26] despite the fact that DLS cannot define the concentration of the vesicles and cannot provide biochemical data or information about the origin of EVs [9, 27].

Although DLS can sensitively measure the size of exosomes and microvesicles with sizes ranging from 1 to 6,000 nm, the intensity of the scattered light is proportional to the sixth power of the EVs diameter, causing the larger particles to be brighter, and so the size distribution tends to overestimate the contribution of larger vesicles. Moreover, DLS is known to be sensitive to the density of the particle suspension, and surface structures or ionic interactions of the particles with their surrounding fluid may affect DLS results, which becomes especially important when dealing with cellular or sub-cellular structures and various surface modifications. Therefore, caution must be taken in interpreting the poly-dispersed EVs size distribution [21, 25, 28]. Not to mislead the conclusion, sample condition and analysis parameters should be properly and constantly controlled, and also standard particles or other comparative specimens and control groups may need to be included. As in the case of exosomes derived from a new cell line, where the radius of vesicles that measured by DLS and TEM has not yet been compared, the DLS result may have to be supported by physical measurements such as TEM. However, the overestimation of larger particles by DLS mentioned above can be an advantage. As in this study that used DLS to measure the size and homogeneity of exosomes, for the clinical or pharmacological application that matters the homogeneity of exosomes, initial evaluation of the quality of exosomes with DLS is recommended. Even if the samples contain very little amount of aggregations or impurities, DLS can sensitively detect impurities or aggregations in the samples within 1 minute [8].

With TEM, exosomes are visualized as double layered cup-shaped membrane structure with their diameter ranges between 30 to 100 nm [8, 27, 29]. However, because of the chemical fixation and dehydration process included in TEM preparation steps, the structures of EVs may inevitably be collapsed, resulting the size of the EVs is underestimated [9, 30, 31]. Currently, there is the significant controversy regarding the actual size range of exosomes, which was primarily defined by EM that was undersized [9, 30, 31]. These must be the reason why the radius data of exosomes from DLS and TEM were significantly different causing about 40 nm difference in radius (Fig 5C). This aspect makes researchers think that the true "gold standard" for EVs characterization does not exist [2]. Besides, EM is not quantitative or objective, and the absorption of nanoparticles to the support film or blotting paper cannot be easily controlled [30]. Moreover, EM is unsuited for daily or routine use because of the difficult accessibility [32]. These days, EM is rarely used for exosomes quantification due to limited reproducibility, inefficiency and labor-intensive aspect [7]. In sum, DLS can be a very powerful tool if used carefully while understanding the radius obtained by DLS can be identical or larger than by EM.

In summary, our analysis provides clear evidence that DLS can be used as an efficient, convenient and noninvasive technique to simply assess the homogeneity and size of exosomes derived from fibrosarcoma before taking electron microscopes rather than other techniques like NTA, ATM, or flow cytometry. In addition, it has great significance in that this paper has characterized the properties of exosomes from mouse fibroblast sarcoma, which was previously not well researched.

## Supporting information

S1 Raw images (PDF)
