# Supplementary material for: The characterization of exosomes from fibrosarcoma cell and the useful usage of Dynamic Light Scattering (DLS) for their evaluation
Source: PLoS One. 2021 Jan 26;16(1):e0231994. doi: 10.1371/journal.pone.0231994 (PMC7837462; doi:10.1371/journal.pone.0231994)
Supplement: S1 Raw images — (PDF) [file pone.0231994.s001.pdf]

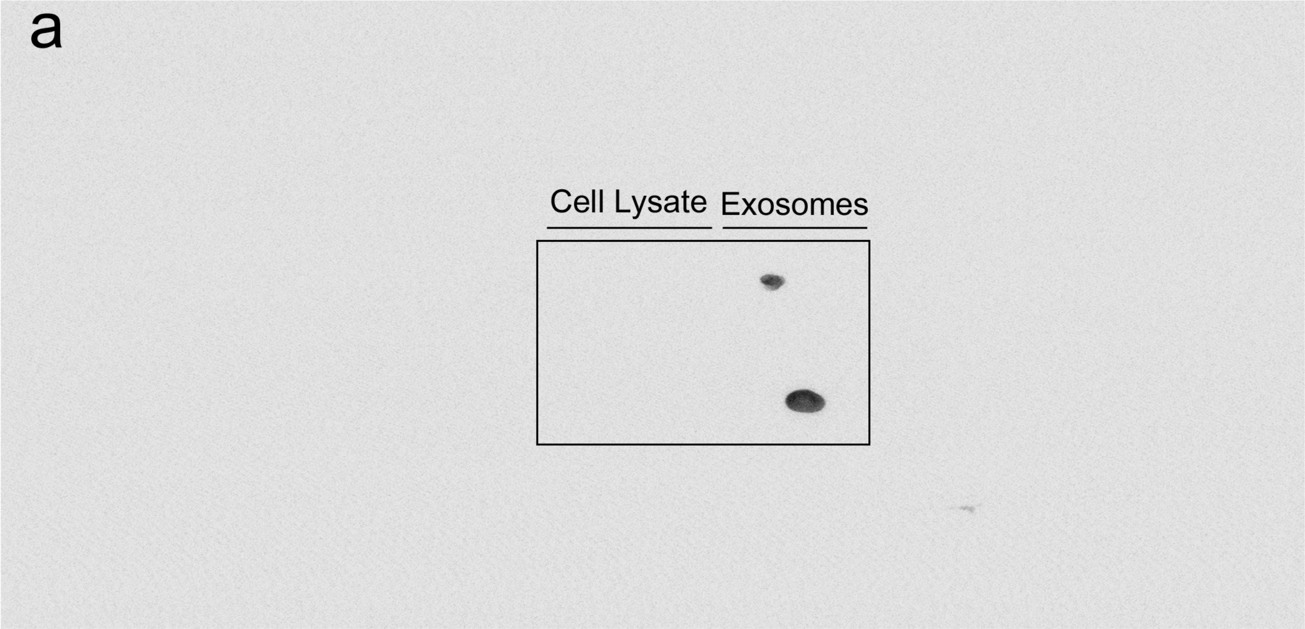

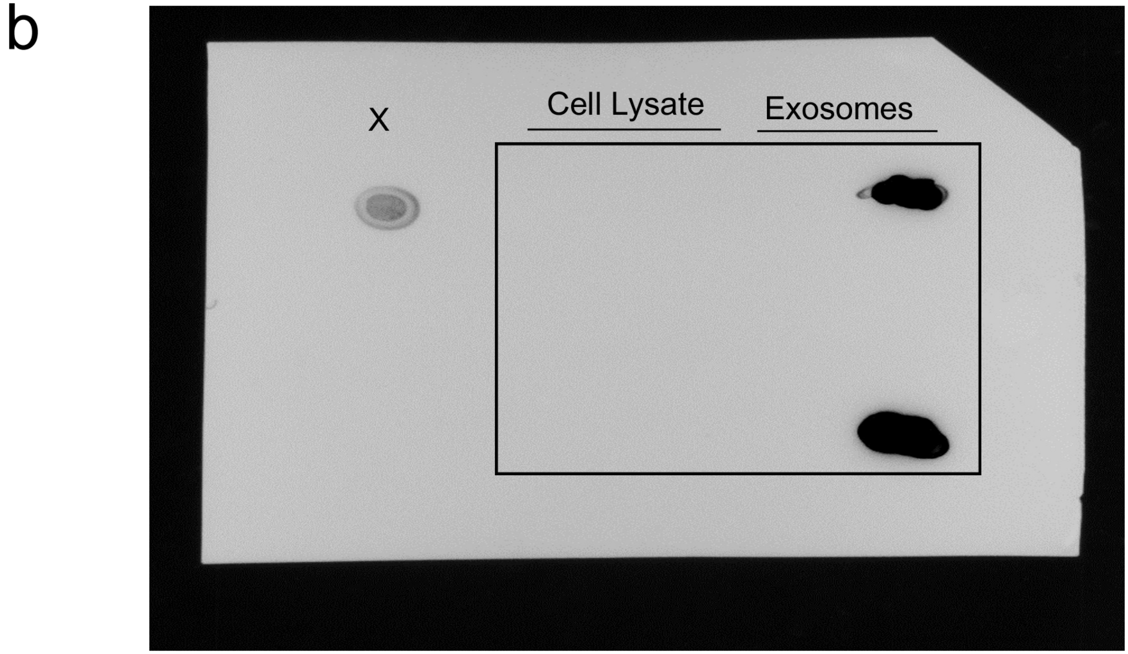

These are raw image of Figure 1., the result of dot blot. The cell lines used in (a) and (b) are WEHI-164 and MDA-MB-231 respectively. ECL solution (Thermo Scientific™ 32106) and illuminator (ATTO Luminograph II) were used to capture the image.

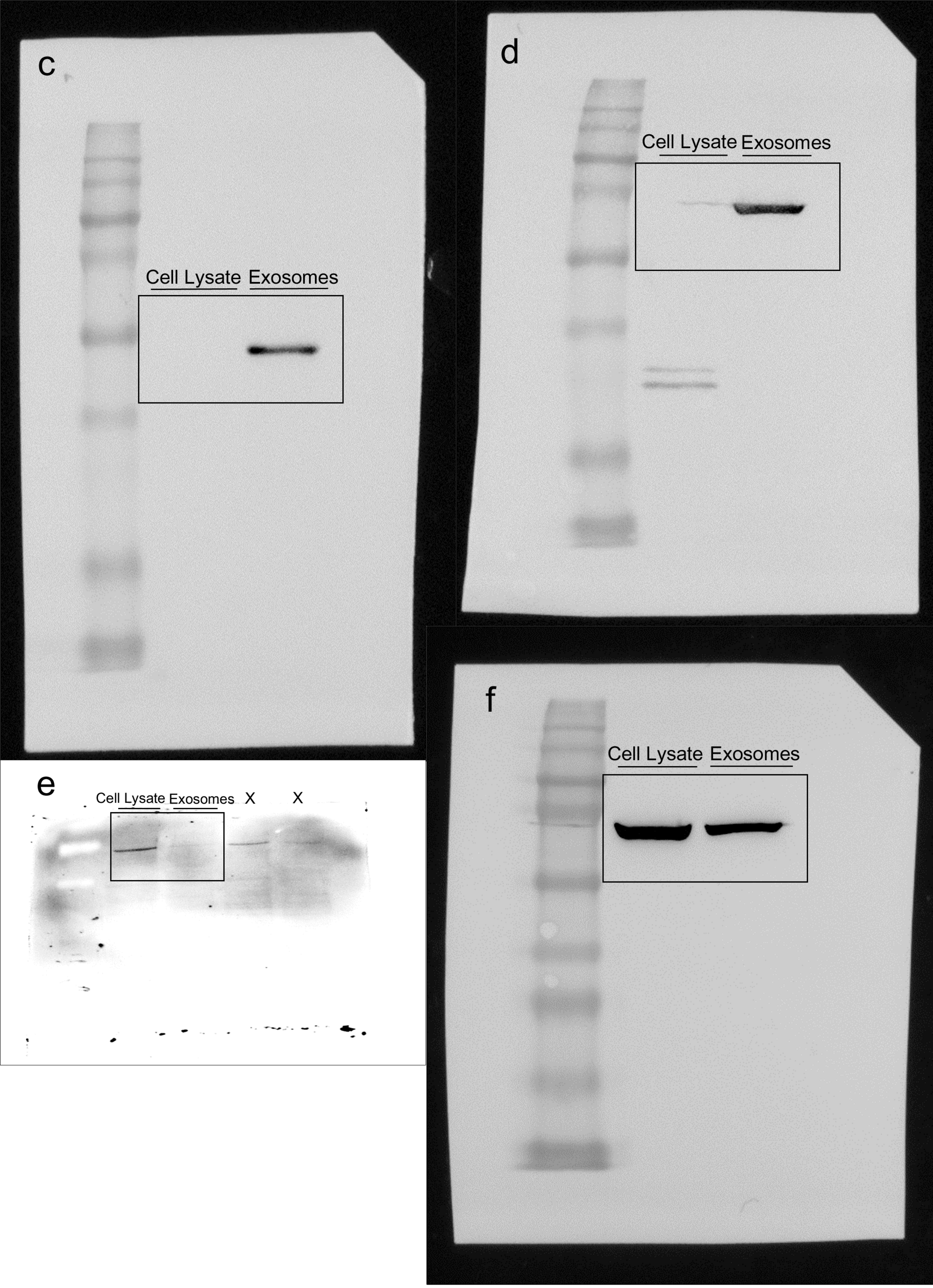

These are raw image of Figure 1.(B). The cell line used in these data is WEHI-164. The antibodies used in (c), (d), (e) and (f) are TSG101, HSC70, Calnexin and GAPDH respectively. ECL solution (Thermo Scientific™ 32106) and illuminator (ATTO Luminograph II) were used to capture the image.

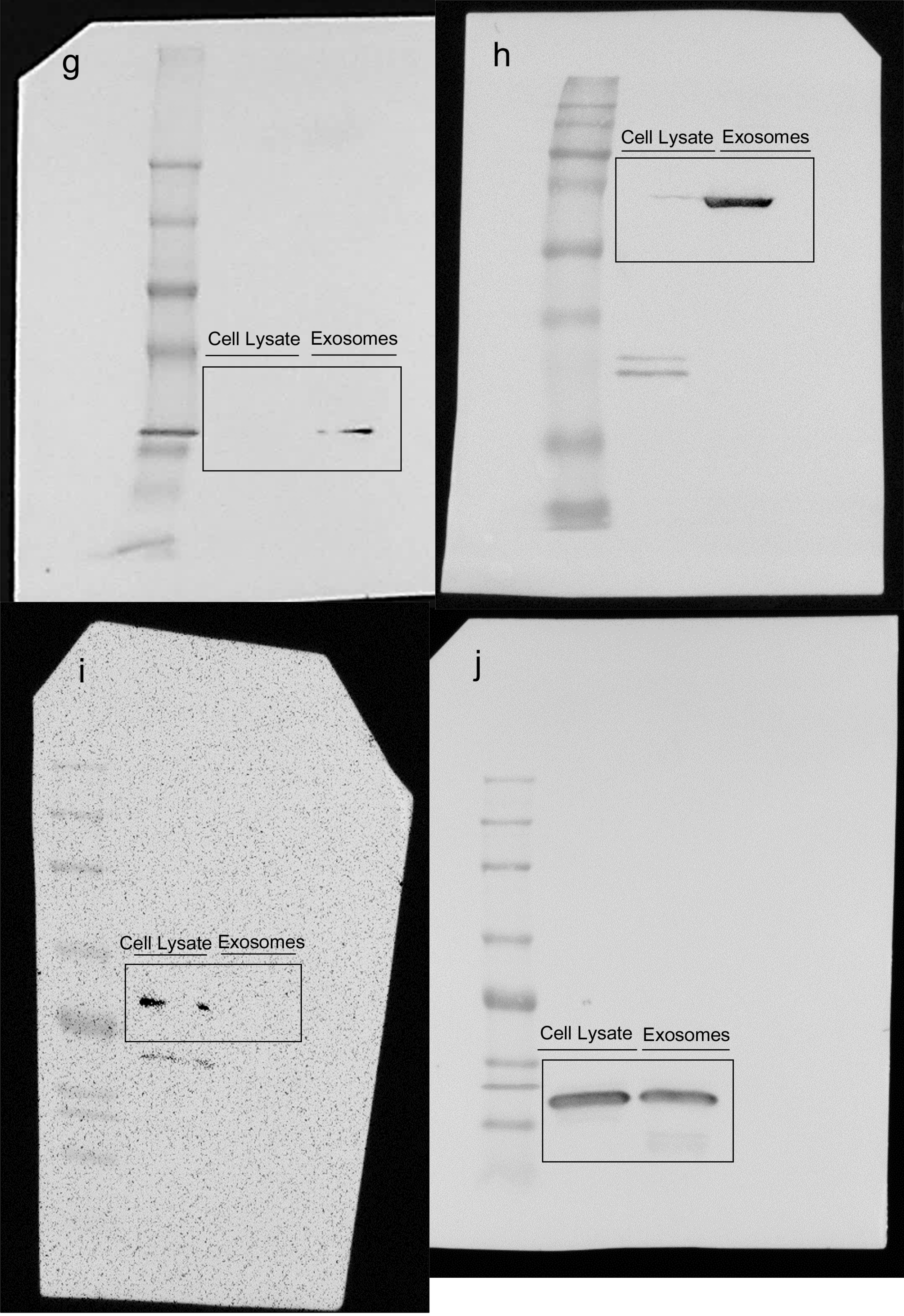

These are raw image of Figure 1.(B). The cell line used in these data is MDA-MB-231. The antibodies used in (g), (h), (i) and (j) are TSG101, HSC70, Calnexin and GAPDH respectively. ECL solution (Thermo Scientific™ 32106) and illuminator (ATTO Luminograph II) were used to capture the image.
